# Supplementary material for: ProteinShader: illustrative rendering of macromolecules
Source: BMC Struct Biol. 2009 Mar 30;9:19. doi: 10.1186/1472-6807-9-19 (PMC2672931; doi:10.1186/1472-6807-9-19)
Supplement: Additional file 1 — ProteinShader program without source code. This compressed file contains the complete ProteinShader program including associated libraries, but no source code. A README.txt file gives an overview of the ProteinShader distribution, and the index.html file in the help subdirectory has directions on getting started with the program as well as a set of tutorials. [file 1472-6807-9-19-S1.zip › ProteinShader-beta-0_9_4-binary/help/tutorials/1AQB.html]

ProteinShader Tutorial: 1AQB


# ProteinShader Tutorial


♦ Home


♦ Download


♦ Getting Started

Choose a Link:

Installation

Running

Troubleshooting

PDB Files


♦ Tutorials

Choose a Link:

List of Proteins

Style Options

Saving Images


♦ General Controls

Choose a Link:

Menu Bar

Keyboard Shortcuts

Mouse Motion

Motion Panel

Antialiasing


♦ Cartoon Controls

Choose a Link:

Control Panels

Selection Panel

Radio Buttons

Decorations Panel

Adding Textures

Color Panel

Visibility Panel

Side Chains Panel


♦ Atom Controls

Choose a Link:

Control Panels

Selection Panel

Radio Buttons

Color Panel

Visibility Panel

Scale Panel

Tiling Panel


♦ Developers

Choose a Link:

Compiling

Running

Code Docs

Developer Links


## Retinol-Binding Protein: Beta-Barrel Structure [PDB:1AQB]

The retinol-binding protein is a beta-barrel structure that is used to hold and
transport the lipid alcohol vitamin A (retinol) through the blood stream
[1, 2].
The three-dimensional structure of this single chain protein has been
solved by x-ray crystallography
[3, 4],
and can be loaded into the ProteinShader program by selecting Open from the
File menu 
and then using the file chooser box to select the 1AQB.pdb file.


## Cartoon-style displays

When the retinol-binding protein is first loaded, it will be displayed as a
pen-and-ink style drawing of ribbons and tubes. To switch to an all tubes
style display as shown in Figure 1A, go to the
Style menu
above the canvas and select Tubes from the Cartoon submenu. Regions of regular
secondary structure such as beta-strands and alpha-helices are shown with a
greater radius (1.5 angstroms) than general loop regions (0.75 angstroms).

To switch from the Figure 1A image to the Figure 1B image, first go to the
Style menu
above the canvas and use the Cartoon submenu to go back to Ribbons and Tubes.
Next, go to the
Decorations subpanel
of the Control Panel on the right side of the canvas and click on the
β-Strands radio button to select all beta-strands. Then select Hatching
1 from the Bend Texture menu, which will change the texture map that is
applied to the middle of each segment of the ribbon that corresponds to an
amino acid.

To fade out the loop regions that connect the beta-strands, go to the menu at
the top right of the Control Panel and use it to change from the
Decorations subpanel
to the 
Cartoon Visibility subpanel. Click on the Loops radio button to select
all loops, and then click on the Translucent button. By default, the button
should be set to use 75% translucency, but the value can be changed by using
the slider control below the button.

To switch from the Figure 1B image to the Figure 1C image, go to the
Orientation menu
above the canvas and select Top. The image will be close to that seen
in Figure 1C, but you will have to left click and drag the mouse across the
canvas a little to move the protein into the same view as in Figure 1C.
The original view can always be restored by going to the
Orientation menu
above the canvas and selecting Original. To zoom in on an image, use
the scroll wheel or center mouse button. If the canvas has the focus (because
it was clicked on), pressing the "i" key once will zoom the camera in
by 5 angstroms, while the "o" key will zoom the camera out by 5
ansgstroms.


|  |  |  |
| --- | --- | --- |
| **A** | **B** | **C** |

**Figure 1.** Pen and ink style illustrations of the retinol-binding protein.
(A) Side view of this beta-barrel structure shown as tubes with halftoning.
(B) Same as in part A, but with the beta-strands and loops shown as ribbons,
and with the loop regions set to 75% translucency. (C) Same as in part B, but
with the image rotated to show an open end of the beta-barrel. The larger of
the two tube-shaped helical structures seen in this view is a gamma-helix,
while the smaller is an alpha-helix (gamma-helices are represented the same
way as alpha-helices in a cartoon-style display).
The images were captured as described in the
Saving Images
section of the tutorials main page.


## Space filling style displays

To display a space filling style view of the retinol-binding protein,
it might be a good idea to first select Open from the
File menu
and reload the 1AQB.pdb file (only to be sure that you are starting with all of
the default settings for the structure). After the protein reloads it will be
displayed again as tubes and ribbons. Now go to the
Style menu
above the canvas and use the Atom submenu to select Space Filling. By default,
the ProteinShader program only shows amino acids when a structure is first
loaded, so go to the
Visibility menu
above the canvas to select Heterogens Allowed and Waters Allowed. After using
the
Background menu
above the canvas to set the background to black, the image should look the same
as in Figure 2A. To see the retinol molecule (a heterogen) buried in the
center of the beta-barrel (Figure 2B), go back to the
Visibility menu
above the canvas to deselect Amino Acids Allowed and Waters Allowed.

To simplify the space filling style display of the retinol-binding protein,
first go back to the
Visibility menu
and reselect Amino Acids. Then use the menu at the top right of the Control
Panel to select the
Atom Visibility subpanel,
and select Model-AA from the Selected menu at the top of the subpanel. Click
on the Invisible button in the bottom half of the panel to make all of the amino
acid atoms invisible. To show just the beta-strands, first click on the
β-Strands radio button. Then go to the Apply To menu in the middle of the
subpanel and select Backbone. Clicking on the Opaque button should now cause
the backbone atoms (N, CA, and C) of the beta-strands to become visible.

To change the color of the beta-strand atoms, go back to the menu at the top
right of the Control Panel and switch from the
Atom Visibility subpanel
to the
Atom Color subpanel.
With the β-Strands radio button still selected, click on the Chooser button
to open a color chooser dialog box and select a shade of green. The image
should now match Figure 2C. To get a better look at the retinol molecule
inside of the barrel, drag the mouse across the canvas to rotate the image
until something similar to Figure 2D is obtained. The retinol can also be
shown in a cartoon-style display of tubes and ribbons, where it will be
shown in space filling form (it did not show up in Figure 1 only because the
Heterogens Allowed item in the
Visibility menu
above the canvas had not been selected yet).


|  |  |
| --- | --- |
| **A** | **B** |
| **C** | **D** |

**Figure 2.** Space filling style display of the retinol-binding protein.
(A) Space filling style display with spheres colored by atom type.
(B) The retinol molecule after the amino acids and waters shown in part A are
set to be invisible. The pink colored sphere is a cadmium ion.
(C) Space filling style display using only the main chain atoms (green) of the
beta-strands (plus the retinol molecule).
(D) Same as part C, but with the image rotated so that one end of the
beta-barrel can be seen. The retinol is contained within the barrel, while
the cadmium ion is outside of the barrel.
The images were captured as described in the
Saving Images
section of the tutorials main page.


## References

1.
Brandon C, Tooze J: **Beta Structures.**
In *Introduction to Protein Structure.* 2nd edition.
New York: Garland Publishing; 1998: 67-88.

2.
Zanotti G, Berni R:
**Plasma retinol-binding protein: structure and interactions with
retinol, retinoids, and transthyretin.**
*Vitam Horm* 2004, **69**: 271-295.

3.
Zanotti G, Panzalorto M, Marcato A, Malpeli G, Folli C, Berni R:
**Structure of pig plasma retinol-binding protein at 1.65 A resolution.**
*Acta Crystallogr, Sect D,* 1998, **54**: 1049-1052.

4.
**Retinol-binding protein PDB entry 1AQB**
[http://www.rcsb.org/pdb/explore/explore.do?structureId=1AQB]


---

|  |  |  |  |
| --- | --- | --- | --- |
| Hosted by |  |  |  |

Webmaster: Joe Weber,
joe.weber AT alumni.duke.edu
  
Web site last updated: December 30th, 2008
  
© 2007–2008 Joseph R. Weber
